# DC Bead*M1*™: towards an optimal transcatheter hepatic tumour therapy

**DOI:** 10.1007/s10856-015-5629-6

**Published:** 2015-12-16

**Authors:** Andrew L. Lewis, Matthew R. Dreher, Vincent O’Byrne, David Grey, Marcus Caine, Anthony Dunn, Yiqing Tang, Brenda Hall, Kirk D. Fowers, Carmen Gacchina Johnson, Karun V. Sharma, Bradford J. Wood

**Affiliations:** Biocompatibles UK Ltd, a BTG International Group Company, Lakeview, Riverside Way, Watchmoor Park, Camberley, GU15 3YL UK; Center for Interventional Oncology, Department of Radiology & Imaging Sciences, Clinical Center, National Institutes of Health, Bethesda, MD USA; Children’s National Medical Center, 1630 Euclid Street NW#1, Washington, DC USA

## Abstract

Clinical use of DC Bead™ loaded with doxorubicin (DEBDOX™) or irinotecan (DEBIRI™), for the treatment of primary and secondary tumours of the liver respectively, is showing great promise. Recently there has been a tendency to select smaller bead size ranges to treat tumours in an effort to allow more drug dose to be administered, improve tumoural penetration and resultant drug delivery and tumour coverage. Herein we describe the development and performance characterisation of a new DC Bead size range (DC Bead*M1*^TM^, 70–150 μm) capable of an increased bead delivery in the distal vasculature, corresponding to greater tumour coverage and drug dose delivered. Both unloaded and drug loaded DC Bead*M1* were shown to have a greater density of distal volume of penetration although the ultimate distal level of penetration was the same as that of the 100–300 µm beads in an in vitro penetration model. Elution of doxorubicin was slower than irinotecan elution, but it was similar when comparing the same drug elution from 70 to 150 µm compared to 100–300 µm beads. Radiopaque versions of 70–150 and 100–300 µm beads were prepared in order to evaluate distribution ex vivo using µ-CT and doxorubicin distribution using epifluorescent microscopy. Liver distribution of the radiopaque versions of the beads was shown to be more distal and efficient at filling smaller vessels with the DC Bead*M1* and correspondingly more beads were found per vessel histologically with a larger area of drug coverage with the smaller size range. This study indicates that the smaller (70–150 μm) beads should permit an increased dose of drug to be administered to both hypervascular and hypovascular tumours as compared to 100–300 µm beads.

## Introduction

Transarterial chemoembolisation (TACE) has been practiced in patients for over 3 decades [1, 2] and describes the targeted delivery of chemotherapeutic agents followed by embolic material(s) to hypervascular liver tumors through a catheter placed in the tumor supplying arteries [3]. Although there is no worldwide standard technique [4], conventional TACE (cTACE) is most often performed by emulsifying chemotherapeutics with Lipiodol^®^ and then infusing this emulsion through the catheter prior to delivery of the embolic material. More recently, drug-eluting bead TACE (DEB-TACE) has emerged as an alternative to cTACE [5]. In DEB-TACE, calibrated size ranges of embolic microspheres are loaded with a drug and then delivered in a single image-guided step. Although use of DEB-TACE has recently increased, clinical practice is still evolving with regard to technique and material choice.

The most commonly used [4] and well characterised DEB is DC Bead, which is a microspherical embolisation device composed of acrylamido polyvinyl alcohol–*co*–acrylamido-2-methylpropane sulfonate capable of loading and eluting positively-charged drugs such as doxorubicin or irinotecan via an ion exchange process [6–11]. TACE using DC Bead loaded with doxorubicin (DEBDOX) has become a popular treatment option for patients with intermediate stage hepatocellular carcinoma (HCC), a highly vascular liver tumour [12]. The first phase I/II DEBDOX studies of Varela et al. [13] and Poon et al. [14] involved a dose-escalation phase and used DEBs measuring 500–700 µm in diameter. This particular size range was a consequence of the direct translation of the embolic agent size historically used to treat these patients using cTACE methods, which often used non-spherical embolic agents such as polyvinyl alcohol (PVA) particles to complete the embolisation after delivery of the chemotherapy emulsion.

The choice of embolic agent size is an important consideration and should be suited to the physicians’ desired level of occlusion, but in addition to size, embolic material composition must also be considered as compressibility of microspheres plays a role in the level of occlusion [15, 16]. Whilst the doxorubicin dosage administered in clinical practice has remained consistent at 100–150 mg per-treatment, there has been a subtle migration to using progressively smaller DEB sizes. This is illustrated in the published literature, such as the first PRECISION I and II studies which used solely 500–700 µm DC Bead [13, 14], with the later randomized phase II trial PRECISION V [17] and the study of Dhanasekaran et al. [18] which recommended use of 300–500 µm and 500–700 µm DC Bead; and those of Malagari et al. [19–21] and Reyes et al. [22] for instance, which used 100–300 µm and 300–500 µm sized products in treatment of HCC. More recent consensus recommendations suggest use of 100–300 µm DEBs [23].

In addition to DEBDOX, the combination of irinotecan with DC Bead (DEBIRI) is an attractive locoregional therapy for some patients with liver metastates from colorectal cancer (mCRC). DEBIRI has shown benefit in patients for whom systemic chemotherapy options have failed and in whom surgical resection is not possible [24–26], as well as those in whom tumor shrinkage is required to allow surgical resection i.e., surgical down-staging and neo-adjuvant therapy [27]. Irinotecan is chosen over doxorubicin in treatment of mCRC due to its common use in an effective systemic combination chemotherapy regimen called FOLFIRI [28]. Although mCRC may be hypervascular relative to the liver, these tumors tend to be less vascularized than HCC and this difference in vascularity can limit the volume of DEBs and irinotecan that can be delivered to the tumour using DEBIRI. Moreover, mCRC often results in multiple and diffuse tumours in the liver requiring a more proximal catheter placement into the right or left hepatic artery followed by a lobar infusion of DEBIRI [26]. These factors motivated the preference of 100–300 µm beads for treatment of mCRC [29], as this was the smallest size commercially available at the time of study initiation for DEBIRI and more often allowed for a greater volume of beads and irinotecan to be delivered to the patient.

The desire for improved penetration must be balanced with potential safety concerns associated with use of very small microspheres. There have been isolated cautionary reports of potentially fatal complications from non-target embolisation following the use of small embolic agents, such as 40–120 µm sized trisacryl microspheres or 40 µm polyphosphazene coated PMMA microspheres [30, 31]. However, it is now generally accepted that appropriate use of careful angiographic analysis for shunting if using small deformable hydrogel microspheres can ameliorate this safety concern associated with the use of smaller devices. A smaller DEB has therefore been developed for use in DEBDOX or DEBIRI, which might provide the benefits of a smaller size range including a greater distal penetration, access to tumour regions perfused with small diameter blood vessels, and provide a higher dose of drug delivered to the target tissue, whilst minimising the associated potential safety risks. This overview compares the in vitro and in vivo performance characteristics of 70–150 µm DEBs with the established DEB size ranges and discusses some of the outcomes from its use in clinical practice.

## Materials and methods

The different bead size ranges were subjected to drug loading and elution studies using both doxorubicin and irinotecan, and the effects of drug loading on bead size and penetration potential evaluated. In order to appreciate the differences in bead distribution in tissue, radiopaque versions of the beads were used to render them visible under X-ray imaging.

### Bead materials

DC Bead *M1* (70–150 µm) and DC Bead (100–300 µm, 300–500 µm, and 500–700 µm) (Biocompatibles UK Limited, a BTG International group company Farnham, UK) are composed of acrylamido polyvinylalcohol-*co*-acrylamido-2-methylpropane sulfonate. Vials containing 2 mL of sedimented bead volume were used for in vitro characterization. To enable in vivo bead distribution studies to be performed and imaged using μCT, radiopaque versions of DC Bead*M1* (radiopaque 70–150 µm) and DC Bead 100–300 µm (radiopaque 100–300 µm) were prepared as previously described [32]. Briefly, beads were lyophilised and incorporated with iodized oil and doxorubicin using a previously reported process [33].

### Bead drug loading

Two mLs (one vial) of 70–150 and 100–300 µm beads were loaded with either 50 mg/mL or 75 mg/mL of doxorubicin hydrochloride (Dabur, India), or 100 mg/mL of irinotecan hydrochloride trihydrate (Dabur, India) via an ionic exchange mechanism. The packing solution was removed from the vial to leave a slurry of beads to which a sterile water drug solution was added and drug uptake determined by measurement of the depleted loading solution at set time intervals by UV–Visible spectrophotometry as previously reported [9, 10]. The resulting drug eluting beads (DEB) loaded with doxorubicin or irinotecan were suitable for in vitro characterization studies.

### Bead sizing

Bead sizes were measured using a BX50 microscope and a ×10 dry objective. (Olympus UK Ltd, Essex, England). To verify the eyepiece graticule used to measure the beads, a calibrated graticule (Graticules Ltd, Kent, England) was placed on the microscope stage and the eyepiece divisions verified. A monolayer of each type of bead (fully hydrated in 0.9 % saline) was placed in a Petri dish on the microscope stage. Using the ×10 objective the diameter of 200 individual beads was measured using the eyepiece graticule. The microscope stage was moved systematically across the sample to ensure no repeat measurement of individual beads. The bead sizing data was entered into a spreadsheet and the data graphs generated using Prism 6 (GraphPad Software, Inc., La Jolla, CA).

### Bead penetration studies

**Fig. 1:**
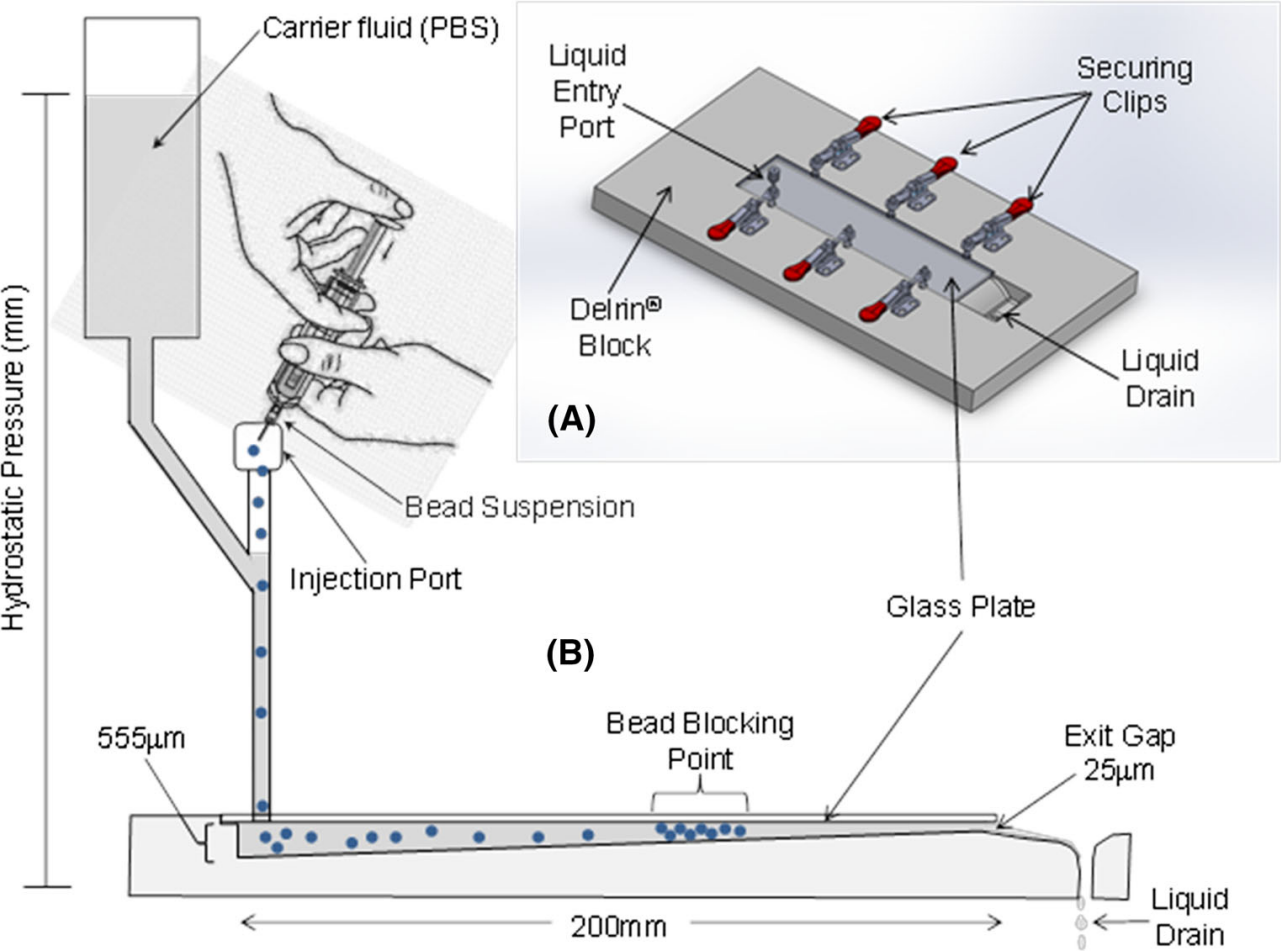
Penetration model schematic. Beads are introduced at the wide end of the taper and flow under the hydrostatic pressure until they block in a band commensurate with their size and mechanical properties

Figure 1 shows a schematic of the experimental set-up of an apparatus designed to measure bead penetration potential. A cut-out section of tapering depth was milled into a block of Delrin^®^ and a glass plate secured over the cut-out section to create a wedge geometry with a depth of 555 µm at one end and 25 µm at the other. The carrier fluid (saline) was held in a reservoir and connected to the plate assembly via an entry port at the wider end of the plate assembly; the pressure under which the fluid flows was controlled by the hydrostatic pressure created when the reservoir was raised 54.4 cm above the penetration device resulting in ~40 mmHg to emulate circulatory blood pressure in appropriately sized vessels (hepatic pressure varying from 90 mmHg in the hepatic artery to 10 mmHg in the sinusoids and the beads generally residing in vessels mid-size to these extremes [34] ).

A bead slurry of between 0.1 and 0.5 mL of sedimented beads (depending on the size being analysed) was introduced via an injection port into the carrier fluid line, whereby the fluid flow (under 40 mmHg hydrostatic pressure) carried the beads into the plate assembly until they were physically constrained by their size and could not migrate further into the narrowing gap. A picture of the plate assembly was captured using a digital camera and the bead blocking point and distribution of beads throughout the plate assembly noted.

### Drug elution using pseudo-sink conditions

Amber jars each containing 500 mL of PBS with a magnetic stir bar were placed in a heated water bath which was on the top of two magnetic stirrer plates. The temperature of the PBS elution medium was controlled at 37 °C by pre-equilibrating the system overnight. Before elution, the drug loaded microsphere samples were brought up to room temperature from refrigerated conditions, and the depleted solution was removed completely. 25 mL of PBS from the elution jar was taken to rinse all the drug-loaded beads into the elution jar. Time zero was defined as when the first sample was placed into the elution media. At each sampling time-point, a 200 mL volume of PBS medium (except for 5 mL at the first sampling time point only) was removed from the jar through a cannula filter, followed by adding back the same volume of fresh PBS to ensure pseudo-sink conditions were maintained. From this withdrawn aliquot, approximately 5–10 mL of eluted sample was used for UV analysis. Absorbance of the eluted sample was measured at 483 nm and 369 nm on a UV–Vis spectrophotometer for doxorubicin and irinotecan respectively.

### Demonstration of bead distribution in vivo as a function of size

All animal studies were conducted under an animal use protocol approved by an NIH Animal Care and Use Committee. VX2 tumours were propagated as described by Ranjan et al. [35]. Briefly, VX2 was propagated in the hind limb of donor rabbits as a single cell suspension. Fragments of these tumours were percutaneously injected into a liver under ultrasound guidance and monitored with ultrasound for 2–3 weeks until a VX2 hepatic tumour of diameter greater than 1 cm in any dimension was established [36].

This rabbit Vx2 intrahepatic tumor model was used to compare the penetration of 70–150 μm with 100–300 μm radiopaque beads after transcatheter embolisation. After induction of anesthesia with ketamine (20 mg/kg; Bedco, St. Joseph, MO), Acepromazine (0.75 mg/kg; Bedco), and Glycopyrolate (1.2 mg; Baxter Healthcare, Dearfield, IL), animals were maintained under general anesthesia with isoflurane. Ketoprofen (5 mg; Fort Dodge Animal Health, Fort Dodge, IA) was given intramuscularly during induction to provide additional analgesia. Femoral access and catheter placement in the proper hepatic artery was conducted with a 3F sheath (Cook, Bloomington, IN) and a 2.4 Fr microcatheter (Terumo, Somerset, NJ) under fluoroscopic guidance. Routine fluoroscopy was conducted using the 9900 Elite Digital Mobile Super C-arm (GE Healthcare/OEC Medical Systems, Inc., Waukesha, WI). Radiopaque beads were mixed 1:20 in saline/Isovue 300 contrast and delivered under fluoroscopic monitoring at a rate of approximately 1 ml/min (including agitation time) until an endpoint of stasis in the proper hepatic artery was angiographically determined. Fluoroscopic images of the targeted organ were obtained intermittently throughout the embolisation and the animal was euthanised 5 min after the completion of embolisation. Following euthanasia, liver and tumor tissue was harvested, placed in formalin and stored at 4 °C prior to ex vivo analysis.

Harvested liver tissue was imaged on 256 slice CT (Philips Healthcare, Best, The Netherlands) with the following settings: 465 mAs, 80 keV, 1 mm thickness, 0.5 mm overlap (typical clinical abdominal scan without contrast) and also 625 mAs, 120 keV, 0.67 mm thickness, 0.335 mm overlap (internal auditory canal (IAC) scan). To resolve individual radiopaque beads within tissue an Inveon microCT (Siemens Preclinical Solutions, Knoxville, TN) was used with the following settings: 31 µm resolution, 80 keV, tube current of 380 µA and a 2 mm Aluminum filter. Images are displayed as 3-dimentional renderings and maximum intensity projections (MIPs) with an arbitrary window and level to maximize contrast.

The data shown for the swine liver embolisation in the discussion were generated according to the methods described in Dreher et al. [37].

### Statistics

Statistical analysis was performed with GraphPad Prism 6.05 (GraphPad Software Inc., La Jolla, CA). Bead size distributions were analysed with descriptive statistics using a relative frequency distribution with a pre-defined bin width. Drug elution was analysed for half-life using first-order (irinotecan) or second order (doxorubicin). Maximum bead penetration was analysed with a two-way ANOVA with factors bead size and drug loading (post hoc analysis with Tukey’s multiple comparison test). All P-values were two-sided and values <0.05 were considered statistically significant.

## Results

### Drug loading characteristics

The rate of drug uptake is known to increase with a decrease in the size of the beads due to an increased surface area to volume [8]. Drug loading of the 70–150 µm range was observed to be only slightly faster than the 100–300 µm size range for doxorubicin and very similar for irinotecan (see Table 1).

**Table 1:** Comparison of drug loading times for DC Bead*M1* and DC Bead 100–300 µm

Loading time (mins)		30	60	120
Drug loading	Bead size range (µm)	% Drug loaded		
37.5 mg/mL Dox	70–150	99.8	100.23	N/A
37.5 mg/mL Dox	100–300	97.89	100.22	N/A
50 mg/mL Iri	70–150	97.5	98.0	99.9
50 mg/mL Iri	100–300	97.1	98.5	100.01

### Bead size

**Fig. 2:**
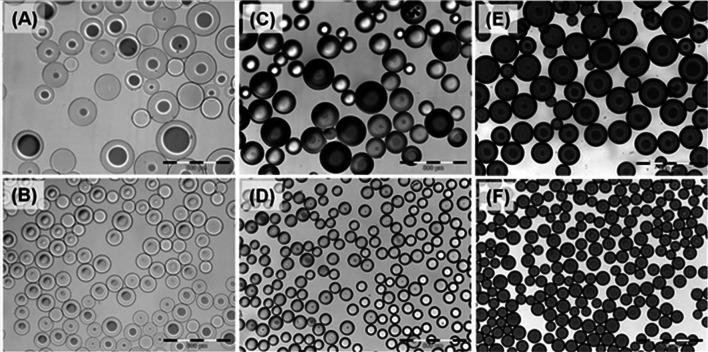
Representative images of 100–300 µm (*top row* **a**, **c**, and **e**) and 70–150 µm (*bottom row* **b**, **d**, and **f**). Bland beads (**a** and **b**), irinotecan loaded beads (**c** and **d**) and doxorubicin loaded beads (**e** and **f**) appear similar except the smaller size range is more uniform and smaller in diameter (*scale bar* each division 100 μm, total length 500 μm)

**Fig. 3:**
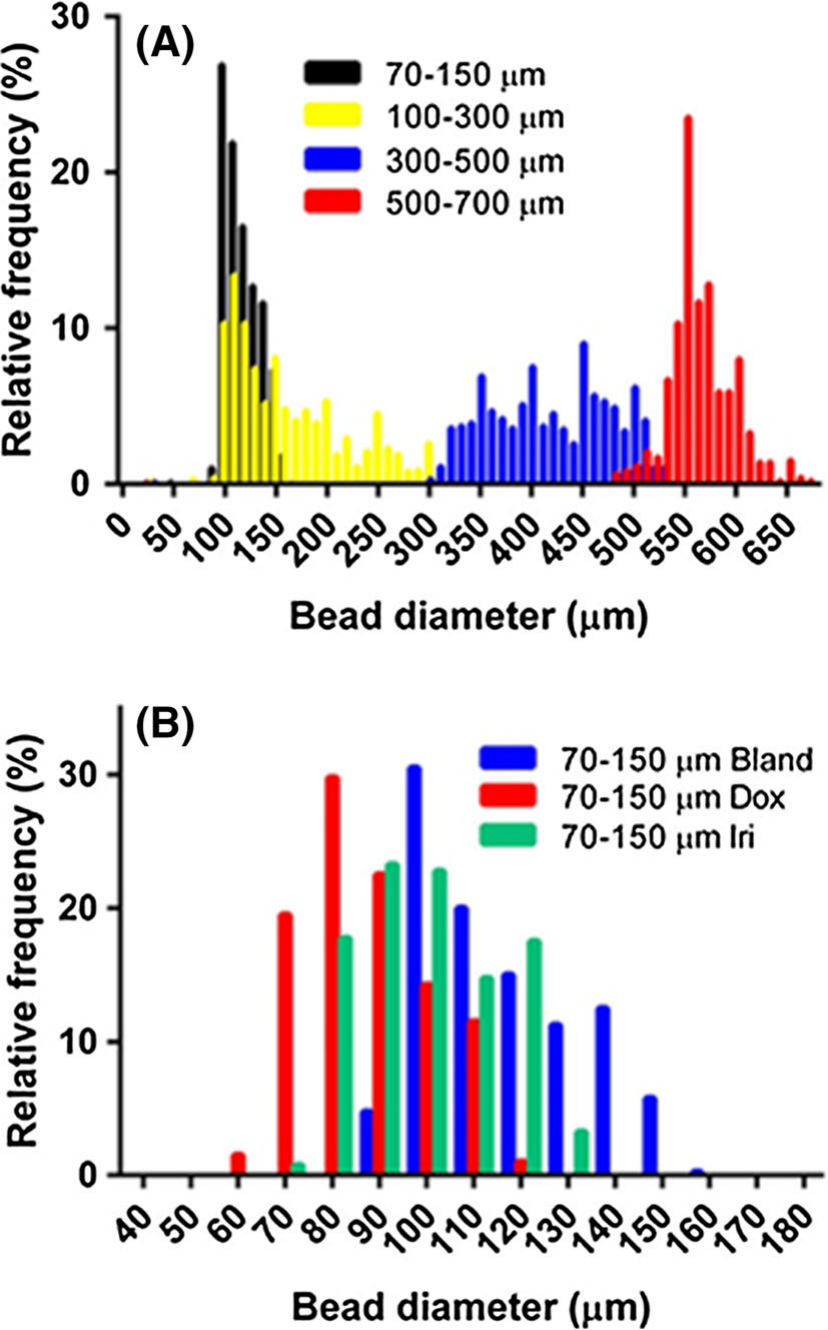
Bead size distribution for various bead size ranges and with drug loading. **a** Bead size distribution in 0.9 % saline shown as a histogram for all DC Bead sizes (n = 1000). **b** Average bead diameters of DC Bead*M1* with 37.5 mg/mL doxorubicin (Dox) and 50 mg/mL irinotecan loading (n = 400)

The smaller bead size (70–150 µm) was obtained by additional sieving to remove the larger bead size fraction found in the 100–300 µm size range as shown in Figs. 2 and 3. The beads appear similarly smooth and spherical in shape but the 70–150 µm is more uniform in bead size distribution. Both bead sizes are capable of loading irinotecan (Fig. 2c, d) and doxorubicin (Fig. 2e, f) resulting in a corresponding decrease in the average diameter of the beads due to water displacement and drug interactions [8] as clearly shown in optical micrographs of Fig. 2 and graphically Fig. 3b. For the 100–300 µm beads there is a percentage decrease in the average size in the range of 7, 12 and 24 % when loaded with either 25 mg/mL doxorubicin, 37.5 mg/mL doxorubicin or 50 mg/mL irinotecan respectively. For the 70–150 µm beads the percentage decrease is relatively greater at 14, 25 and 28 % respectively for the same drug doses (see Fig. 3b).

### *In vitro* penetration properties

Bead penetration was quantified with a custom penetration model, shown in Fig. 1, which created a wedge geometry that decreased in gap size in the direction of flow. Interestingly, the more narrow size distribution in the 70–150 µm beads results in a more uniform leading edge while the other bead sizes have a more sporadic appearance where some larger beads in the distribution decrease the penetration of subsequent smaller beads. There was a significant effect of bead size and drug loading on the maximum penetration (*P* value <0.05, 2 way ANOVA). Importantly, the average bead penetration was not different for 70–150 and 100–300 μm (*P* value >0.05, Tukey’s) but significant for all other comparisons with 300–500 μm (*P* value <0.05, Tukey’s). Doxorubicin and irinotecan loading significantly increased the penetration for all bead sizes (*P* value <0.05, Tukey’s) except 100–300 μm with irinotecan.

**Fig. 4:**
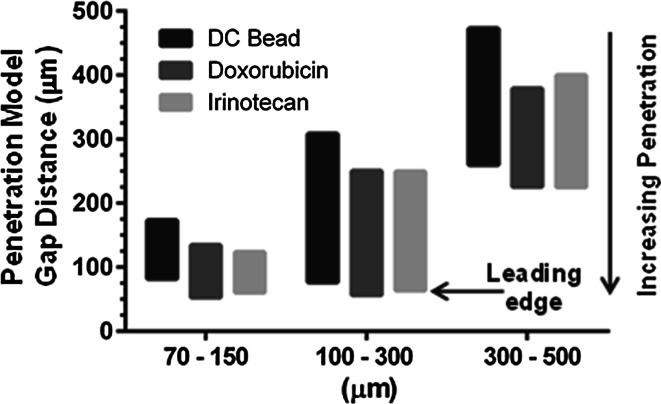
Quantification of in vitro bead penetration (drug loading levels: 37.5 mg/mL doxorubicin and 50 mg/mL irinotecan)

When loaded with drug, as shown in Fig. 4, trends are similar to bland beads with a nearly identical leading edge between size ranges but on average a much greater penetration and uniformity for the smaller size range. These data suggest the reduction in size due to drug loading is balanced by an increase in stiffness, resulting in a similar maximum bead penetration.

### Drug elution characteristics

**Fig. 5:**
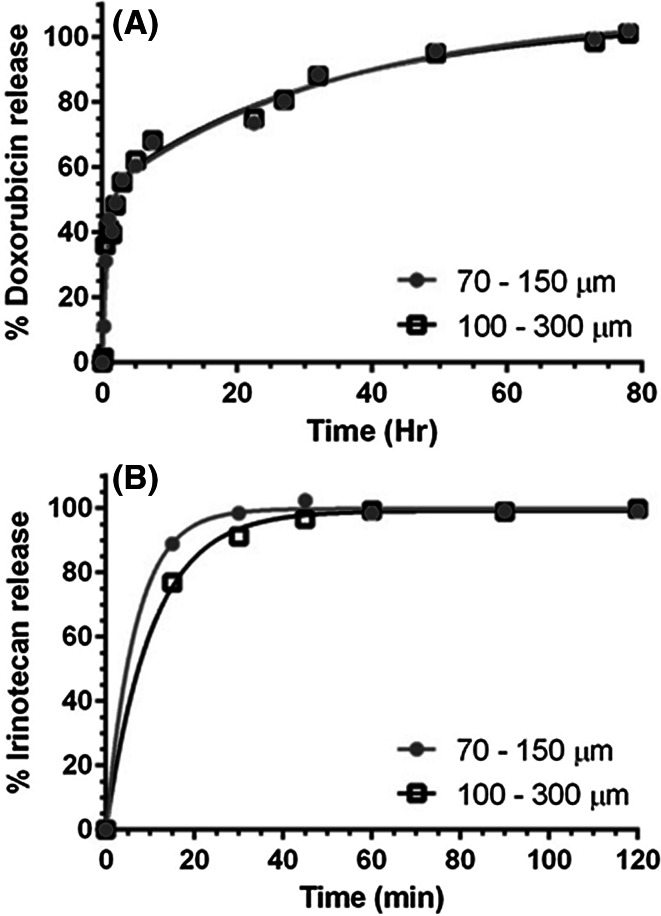
**a** Comparisons of doxorubicin elution (37.5 mg/mL loading) for 70–150 and 100–300 μm size DC Bead sizes (n = 2). **b** Comparisons of irinotecan elution (50 mg/mL loading) for 70–150 and 100–300 μm size DC Bead sizes (n = 2)

Figure 5a shows that under the rapid elution conditions of this in vitro test, complete elution of doxorubicin is achieved over several hours with both the 100–300 µm bead (half-life (time for 50 % elution) = 0.57 h) and 70–150 µm beads (half-life = 0.52 h) eluting at the same rate. Irinotecan elution however, takes only around an hour under the same conditions (half-life = 7.26 min) with the 100–300 µm bead size range eluting marginally slower than the 70–150 µm beads (half-life 4.68 min) (Fig. 5b).

### In vivo bead distribution

**Fig. 6:**
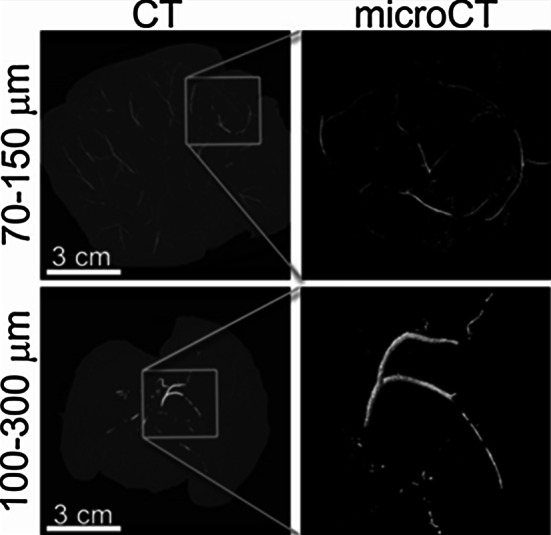
CT and microCT of 70–150 and 100–300 µm radiopaque beads. 70–150 µm beads provide a more distal embolisation and obtain greater tumour coverage. The box in the CT image demonstrates the location of the tumour

In order to gain an appreciation of how the 70–150 µm beads might distribute within the vasculature in vivo, a radiopaque version of this product was prepared and compared to a corresponding radiopaque 100–300 µm product, as shown in Fig. 6. 70–150 µm beads result in a more distal embolisation; reaching the periphery of the rabbit liver tissue. In contrast, 100–300 µm are located more central in the liver. Both beads reached the tumour but the 70–150 µm provided greater coverage of the tumor periphery and some penetration into the tumor core.

## Discussion

### Study findings and the state of the art

Most of the published literature describes the use of DC Bead in sizes ranges of 100–300 µm, 300–500 µm and 500–700 µm. Regardless of size, the use of this device loaded with doxorubicin to treat HCC has yielded some encouraging 4 [38] and 5 [39] year survival data. From a physical perspective, as the size range becomes smaller, the percent difference between the smallest and largest beads within this population becomes increasingly large (Figs. 2, 3); hence, for the 500–700 µm range there is a 1.4- fold difference but for the 100–300 µm range there is a threefold difference in the sizes of arteries that may be occluded. We hypothesised that if this difference in the 100–300 µm range could be refined to remove the larger sized bead fraction, the resulting DEB would have greater distal penetration. Similar to larger size ranges, the 70–150 µm range was manufactured with a modified reverse suspension polymerisation process but this 100–300 µm product was further sieved in order to remove the higher-end diameter beads, whilst maintaining the lower size specification limit that is identical to the current 100–300 µm size range.

**Fig. 7:**
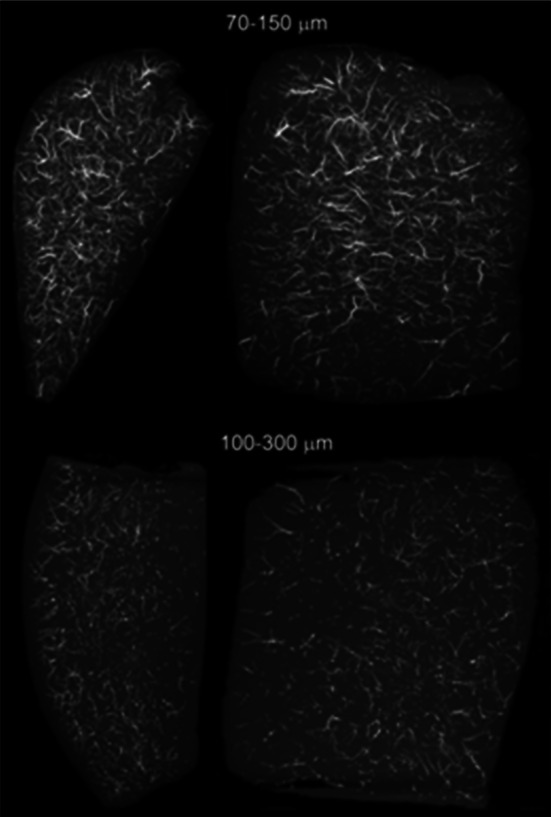
Comparison of the distribution of 70–150 µm (*top*) and 100–300 µm (*bottom*) radiopaque beads in embolised swine liver tissue (sagittal (*left*) and coronal (*right*) views of the tissue sample). Note that despite the same lower limit of bead size, the 70–150 µm bead are able to penetrate down into the tiny vessels at the liver capsule surface, whereas there is a clear bead-free margin at the surface of the sample containing the 100–300 µm beads (unpublished data from the study described in Ref. [37])

In this investigation drug loading of the 70–150 µm range was observed to be faster than for the 100–300 µm size range for both doxorubicin and irinotecan (see Table 1), as anticipated by the overall increase in surface area to volume ratio that arises as a consequence of its smaller average bead diameter. Prior to clinical evaluation, thorough bench testing was required in order to determine if the physicomechanical properties of the 70–150 µm beads were similar to conventional 100–300 µm bead size range. From data such as that shown in Fig. 4 for instance, it can be seen that both size ranges have the potential to penetrate to the same maximum level of occlusion, demonstrating that the smallest bead fraction of either size range are both similar in size and compressive nature (somewhat addressing potential safety concerns). Clearly, the loading of either drug decreased the average bead diameter, but resulted in a concomitant increase in the resistance to compression [8, 10] that culminated in a marginal increase in distal penetration. However, the larger size range of 100–300 µm results in a greater distribution in the level of penetration. This test demonstrates the ability of the beads to compress, deform and occlude levels below their nominal lowest bead diameter; this however, is not directly translatable to the situation in vivo, as in this in vitro penetration model the beads are compressed in a quasi-uniaxial direction between two flat surfaces as opposed to being deformed within a cylindrically-shaped vessel. Furthermore, in vivo a large bead from the 100–300 µm size range may prematurely occlude a blood vessel before the smaller beads within the size distribution have the opportunity to penetrate to their nominal size. The tighter size distribution of 70–150 µm limits the likelihood of in vivo premature occlusion. This is demonstrated in this study (Fig. 6) in which we have investigated the distribution in a tumour-bearing rabbit liver, where we see that the 100–300 µm beads have a tendency to occlude and accumulate in the larger, proximal and more central hepatic arteries, whereas the 70–150 µm beads are seen clearly occupying the smaller, more distal and peripheral arteries and those surrounding the tumour (Fig. 7).

**Fig. 8:**
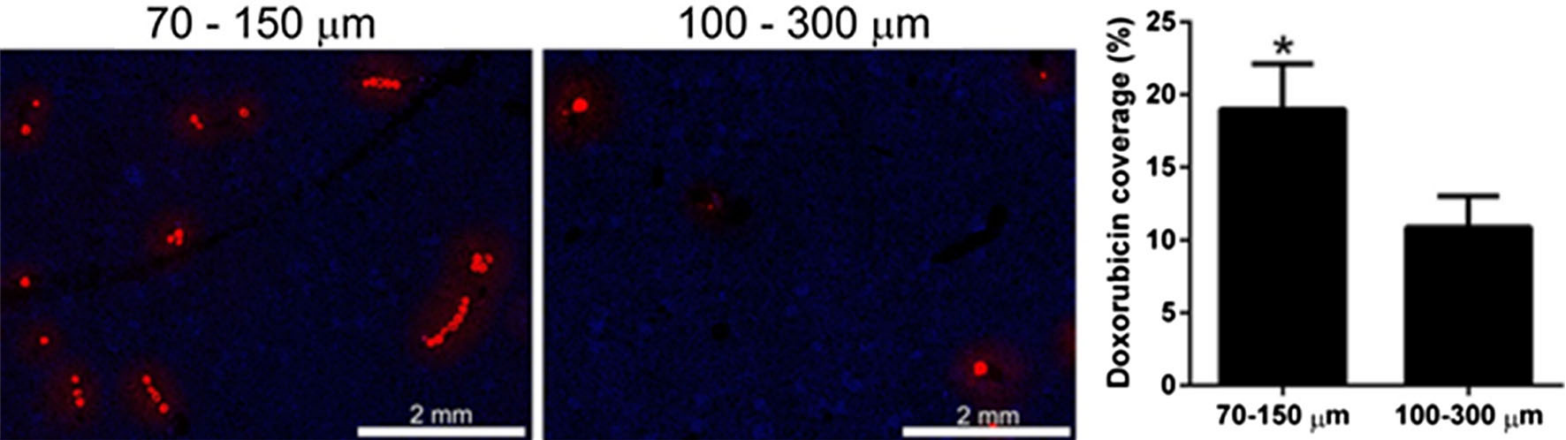
Epifluorescent microscopy of liver tissue sections embolised with 70–150 µm versus 100–300 µm doxorubicin loaded DC Bead showing increased bead number, spatial distribution and drug penumbra (*left*) and the corresponding comparison of the percentage drug coverage (*right*, unpublished data from [37])

Under pseudo-sink elution conditions, it was expected to observe a relatively slow elution of doxorubicin from the beads due to strong drug-bead and drug–drug interactions [7], whilst irinotecan release should occur over a much shorter period. Doxorubicin elution rates were similar for both 70–150 µm and 100–300 µm beads when measured under pseudo-sink conditions which is consistent with data previously presented on the comparison of the systemic pharmacokinetics of doxorubicin release from the 100–300 µm bead and 70–150 µm beads in a rabbit VX2 tumour model performed at Johns Hopkins University [40], that showed blood plasma levels to be equivalent and low compared to historical values generated for cTACE [41]. They reported a similar finding for the first 10 patients treated with the 70–150 µm beads compared to historical data from 500 to 700 µm beads [42], confirming that in vivo there is less difference in the systemic exposure of doxorubicin between different sized beads than that predicted by in vitro methodologies [43]. The Hopkins VX2 study also showed that both the number of beads present and the doxorubicin concentration in the tumour were 2.6-fold greater for the 70–150 µm beads compared to 100–300 µm beads. These data are consistent with the work of Dreher et al. that demonstrated a ~twofold increase in drug coverage using a swine liver model ( [37], Fig. 8). The 70–150 µm beads therefore appear to offer the enhanced locoregional drug delivery benefits of low systemic drug exposure [13] with greater tumour concentrations.

As there is a strong desire by treating physicians to consistently deliver the full planned bead volume and associated drug dose to often smaller and/or less vascular tumours, the use of DEB sizes smaller than 100–300 µm should allow access to a greater portion of the targeted vascular volume and may achieve this goal, while at the same time lead to a more distal embolisation. This rationale is supported by data obtained from resected liver lesions following DEBDOX which suggest that most of the 100–300 µm beads are confined to the tumour periphery [44], the previously mentioned preclinical studies which demonstrated the more distal occlusion and associated greater drug coverage for 70–150 µm beads [37] and our data presented here on comparative beads distributions in a VX2 tumour model. More recently in the Spreafico study of HCC patients undergoing DEB-TACE with 70–150 µm beads as a bridge to transplant, they demonstrate histologically that these small beads can penetrate deep within the tumour, even in small nodules where the vasculature is not as well developed [45]. In a study reported by Kelly et al. comparing 70–150 µm beads to 100–300 µm beads for the delivery of irinotecan to patients with hepatic metastatic disease, they demonstrated that when using the 70–150 µm beads there was less premature vessel occlusion and hence more of the drug dose administered, together with a lower adverse event rate (Table 2, [46] ); a finding confirmed in a recent propensity score matching analysis of small versus large DEBIRI [47]. These data validate the bench testing and preclinical data that the 70–150 µm beads have the ability to allow more volume of beads to be delivered before stasis is reached and hence more of the drug dose can be administered.

**Table 2:** Results from a study comparing 70–150 µm beads to 100–300 µm beads for the delivery of irinotecan to patients with hepatic metastatic disease (degree of occlusion determined angiographically) [47]

	N = 303	N = 34
	DC Bead 100–300 µm	DC Bead^*M1*^ 70–150 µm
Dose delivered		
<100 mg	77 %	18 %
100 mg	23 %	82 %
Degree of occlusion (%)		
None	0 %	6 %
Partial	39 %	62 %
Near	26 %	29 %
Complete	35 %	3 %
Adverse events (%)	19 %	6 %

### Review of current clinical safety and efficacy data

Padia et al. recently reported a study of 61 patients with HCC treated with either 100–300 µm (n = 39) or 300–500 µm (n = 22) LC Bead^TM^ loaded with 50 mg of doxorubicin. They found a significantly lower incidence of postembolisation syndrome (PES, one of the commonest side effects of transarterial embolisation and chemoembolisation comprising of fever, nausea/vomiting and pain) (*P* = 0.011) and fatigue (*P* = 0.025) after treatment in the 100–300 μm group (8 and 36 %) versus the 300–500 μm group (40 and 70 %). The mean change in tumour size was similar between the two groups based on WHO and EASL criteria and similar rates of objective response (OR), but there was a trend toward a higher incidence of EASL complete response (CR) with 100–300 versus 300–500 μm beads (59 vs. 36 %; *P* = 0.114) [48]. Such findings have motivated others to assess the 70–150 µm beads to see if even smaller beads improve outcomes further.

Spreafico et al. treated 45 consecutive patients with HCC using 70–150 µm beads and obtained an OR rate [CR + partial response (PR)] of 77.7 % with a median time to best response of 3 months (95 % confidence interval 2–4). In 13 patients, the treatment served as a bridge/downstaging to LT/surgery and pathological analysis of the removed tumors showed that more than 90 % necrosis was achieved in 10 of 28 nodules. The small beads were well tolerated, and the grade 3/4 adverse event rate was low (1 of 65 procedures) [45]. Justaniah et al. conducted a retrospective analysis of three cohorts of HCC patients treated with 100 mg of doxorubicin from two vials of beads of different sizes: 300–500 and 500–700 µm beads (n = 74, large bead group), 100–300 and 300–500 µm beads (n = 33, medium bead group) or 70–150 and 100–300 µm beads (n = 36, small bead group) over a period spanning 2008–2013. They found CR rates for patients treated with small beads were significantly higher at 3 and 6 months than those treated with larger beads [CR 34 vs. 73 vs. 92 % at 3 months (*P* < 0.01) for large, medium and small respectively]. At 9 and 12 months the CR rates were no longer significant due to disease progression (PD) in untreated areas [CR 27 vs. 63 vs. 72 % (P = 0.06) and PD 21, 17 and 15 % respectively]. Heithaus et al. recently reported on midterm outcomes from 94 DEB-TACE sessions using 70–150 µm beads in which disease control (DC = CR + PR + SD) was achieved in 95 % of focal and 96 % of small tumors *versus* 61 % of multifocal (*P* = 0.04) and 55 % of large tumors (*P* = 0.0009). They concluded the treatment was safe with no grade 4/4 complications, 88 % of patients discharged within 24 h and demonstrated antitumoural response and survival benefits [49]. Rao et al. demonstrated the safety and feasibility of performing a DEB-TACE procedure in 31 consecutive patients treated with 70–150 µm beads, as an outpatient procedure with same-day discharge for 88 % of patients given their observations of decreased symptoms of PES [50]. Yet there are reports in some studies with 70–150 µm beads that yielded increased PES relative to 100–300 µm beads (11 vs. 0 %), nausea (33 vs. 14 %), fatigue (50 vs. 14 %) (all *P* > 0.05) although hair loss was significant (39 vs. 0 %, *P* = 0.01) [51]. Thus it is clear that the technique of delivery is very important in determining both outcome and tolerability of the treatment when using smaller drug eluting bead products and more detailed clinical investigation is warranted.

## Conclusions

As clinical experience is mounting for the use of DEB for the treatment of hepatic malignancies, techniques for bead administration (e.g. “embo-light” (a near-stasis end-point but with some residual forward flow) versus stasis [5] ) have advanced along with refinement of drug dose and bead size selection. A number of other DEB technologies have appeared on the market but it would be dangerous to assume similar clinical performance without comparative data, as each has unique chemistry and physical properties that will influence their handling, administration, embolization end-point, rate and extent of drug elution (and hence ultimately drug bioavailability). This evolution of clinical technique and device characteristics relies on the iterative interaction between the bench and bedside. There is sufficient evidence from a variety of animal models [10, 41, 52–55], human PK studies [13, 14] and liver explant evaluations [32, 44, 45, 56, 57] to support the fact that DEBDOX and DEBIRI result in lower systemic drug exposure and higher intratumoural drug concentrations than achieved by hepatic arterial infusion or conventional TACE. Although the optimal procedural conditions for DEB administration however, have not yet been clearly defined, a new DEB size range capable of an increased distal volume (dose) penetration (70–150 µm) offers potential for improved therapeutic outcomes. This smaller and narrower size range provides a relatively similar drug loading and elution profile in addition to the desired consistent distal penetration. The availability of this product and the data generated from its clinical use may start to shed light on the optimum configuration required for transcatheter therapy of hepatic malignancies using DEB.

## References

[1] Yamada R, Nakatsuka H, Nakamura K, Sato M, Itami M, Kobayashi N (1980). Hepatic artery embolization in 32 patients with unresectable hepatoma. Osaka City Med J.

[2] Marelli L, Stigliano R, Triantos C, Senzolo M, Cholongitas E, Davies N (2007). Transarterial therapy for hepatocellular carcinoma: which technique is more effective? A systematic review of cohort and randomized studies. Cardiovasc Intervent Radiol.

[3] Brown DB, Gould JE, Gervais DA, Goldberg SN, Murthy R, Millward SF (2009). Transcatheter therapy for hepatic malignancy: standardization of terminology and reporting criteria. J Vasc Interv Radiol.

[4] Gaba RC (2012). Chemoembolization practice patterns and technical methods among interventional radiologists: results of an online survey. Am J Roentgenol.

[5] Liapi E, Geschwind JF (2011). Transcatheter arterial chemoembolization for liver cancer: is it time to distinguish conventional from drug-eluting chemoembolization?. Cardiovasc Intervent Radiol.

[6] Biondi M, Fusco S, Lewis AL, Netti PA (2012). New insights into the mechanisms of the interactions between doxorubicin and the ion-exchange hydrogel DC Bead™ for use in transarterial chemoembolization (TACE). J Biomater Sci Polym Ed.

[7] Gonzalez MV, Tang Y, Phillips GJ, Lloyd AW, Hall B, Stratford PW (2007). Doxorubicin eluting beads-2: methods for evaluating drug elution and in vitro:in vivo correlation. J Mater Sci Mater Med.

[8] Lewis AL, Gonzalez MV, Leppard SW, Brown JE, Stratford PW, Phillips GJ (2007). Doxorubicin eluting beads - 1: effects of drug loading on bead characteristics and drug distribution. J Mater Sci Mater Med.

[9] Lewis AL, Gonzalez MV, Lloyd AW, Hall B, Tang Y, Willis SL (2006). DC bead: in vitro characterization of a drug-delivery device for transarterial chemoembolization. J Vasc Interv Radiol.

[10] Taylor RR, Tang Y, Gonzalez MV, Stratford PW, Lewis AL (2007). Irinotecan drug eluting beads for use in chemoembolization: in vitro and in vivo evaluation of drug release properties. Eur J Pharm Sci.

[11] Lewis AL (2009). DC Bead(TM): a major development in the toolbox for the interventional oncologist. Expert Rev Med Devices.

[12] Lewis AL, Holden RR (2011). DC Bead embolic drug-eluting bead: clinical application in the locoregional treatment of tumours. Expert Opin Drug Deliv.

[13] Varela M, Real MI, Burrel M, Forner A, Sala M, Brunet M (2007). Chemoembolization of hepatocellular carcinoma with drug eluting beads: efficacy and doxorubicin pharmacokinetics. J Hepatol.

[14] Poon RTP, Tso WK, Pang RWC, Ng KKC, Woo R, Tai KS (2007). A phase I/II trial of chemoembolization for hepatocellular carcinoma using a novel intra-arterial drug-eluting bead. Clin Gastroenterol Hepatol.

[15] Hidaka K, Moine L, Collin G, Labarre D, Grossiord JL, Huang N (2011). Elasticity and viscoelasticity of embolization microspheres. J Mech Behav Biomed Mater.

[16] Verret V, Ghegediban SH, Wassef M, Pelage JP, Golzarian J, Laurent A (2011). The arterial distribution of Embozene and Embosphere microspheres in sheep kidney and uterus embolization models. J Vasc Interv Radiol.

[17] Lammer J, Malagari K, Vogl T, Pilleul F, Denys A, Watkinson A (2010). Prospective randomized study of doxorubicin-eluting-bead embolization in the treatment of hepatocellular carcinoma: results of the PRECISION V study. Cardiovasc Intervent Radiol.

[18] Dhanasekaran R, Kooby DA, Staley CA, Kauh JS, Khanna V, Kim HS (2010). Comparison of conventional transarterial chemoembolization (TACE) and chemoembolization with doxorubicin drug eluting beads (DEB) for unresectable hepatocelluar carcinoma (HCC). J Surg Oncol.

[19] Malagari K, Alexopoulou E, Chatzimichail K, Hall B, Koskinas J, Ryan S (2007). Transcatheter chemoembolization in the treatment of HCC in patients not eligible for curative treatments: midterm results of doxorubicin-loaded DC bead. Abdom Imaging.

[20] Malagari K, Chatzimichael K, Alexopoulou E, Kelekis A, Hall B, Dourakis S (2008). Transarterial chemoembolization of unresectable hepatocellular carcinoma with drug eluting beads: results of an open-label study of 62 patients. Cardiovasc Intervent Radiol.

[21] Malagari K, Pomoni M, Kelekis A, Pomoni A, Dourakis S, Spyridopoulos T (2010). Prospective randomized comparison of chemoembolization with doxorubicin-eluting beads and bland embolization with BeadBlock for hepatocellular carcinoma. Cardiovasc Intervent Radiol.

[22] Reyes DK, Vossen JA, Kamel IR, Azad NS, Wahlin TA, Torbenson MS (2009). Single-center phase II trial of transarterial chemoembolization with drug-eluting beads for patients with unresectable hepatocellular carcinoma: initial experience in the United States. Cancer J.

[23] Lencioni R, de Baere T, Burrel M, Caridi JG, Lammer J, Malagari K (2012). Transcatheter treatment of hepatocellular carcinoma with Doxorubicin-loaded DC Bead (DEBDOX): technical recommendations. Cardiovasc Intervent Radiol.

[24] Aliberti C, Tilli M, Benea G, Fiorentini G (2006). Trans-arterial chemoembolization (TACE) of liver metastases from colorectal cancer using irinotecan-eluting beads: preliminary results. Anticancer Res.

[25] Fiorentini G, Aliberti C, Turrisi G, Del Conte A, Rossi S, Benea G (2007). Intraarterial hepatic chemoembolization of liver metastases from colorectal cancer adopting irinotecan-eluting beads: results of a phase ii clinical study. In Vivo..

[26] Martin RC, Joshi J, Robbins K, Tomalty D, O’Hara R, Tatum C (2009). Transarterial chemoembolization of metastatic colorectal carcinoma with drug-eluting beads, irinotecan (DEBIRI): multi-institutional registry. J Oncol.

[27] Bower M, Metzger T, Robbins K, Tomalty D, Valek V, Boudny J (2010). Surgical downstaging and neo-adjuvant therapy in metastatic colorectal carcinoma with irinotecan drug-eluting beads: a multi-institutional study. HPB (Oxford).

[28] Kamnerdsupaphon P, Lorvidhaya V, Chitapanarux I, Tonusin A, Sukthomya V (2007). FOLFIRI chemotherapy for metastatic colorectal cancer patients. J Med Assoc Thai.

[29] Lencioni R, Aliberti C, de Baere T, Garcia-Monaco R, Narayanan G, O’Grady E (2014). Transarterial treatment of colorectal cancer liver metastases with irinotecan-loaded drug-eluting beads: technical recommendations. J Vasc Interv Radiol.

[30] Bonomo G, Pedicini V, Monfardini L, Della Vigna P, Poretti D, Orgera G (2010). Bland embolization in patients with unresectable hepatocellular carcinoma using precise, tightly size-calibrated, anti-inflammatory microparticles: first clinical experience and one-year follow-up. Cardiovasc Intervent Radiol.

[31] Brown KT (2004). Fatal pulmonary complications after arterial embolization with 40-120- micro m tris-acryl gelatin microspheres. J Vasc Interv Radiol.

[32] Sharma KV, Dreher MR, Tang Y, Pritchard W, Chiesa OA, Karanian J (2010). Development of “imageable” beads for transcatheter embolotherapy. J Vasc Interv Radiol.

[33] Johnson CG, Tang Y, Beck A, Dreher MR, Woods DL, Negussie AH (2015). Preparation of radiopaque drug-eluting beads for transcatheter chemoembolization. J Vasc Interv Radiol.

[34] Khurana I, Circulation R (2006). Textbook of Medical Physiology.

[35] Ranjan A, Jacobs GC, Woods DL, Negussie AH, Partanen A, Yarmolenko PS (2012). Image-guided drug delivery with magnetic resonance guided high intensity focused ultrasound and temperature sensitive liposomes in a rabbit Vx2 tumor model. J Control Release.

[36] Johnson CG, Sharma KV, Levy EB, Woods DL, Morris AH, Bacher JD (2015). Microvascular perfusion changes following transarterial hepatic tumor embolization. J Vasc Interv Radiol.

[37] Dreher MR, Sharma KV, Woods DL, Reddy G, Tang Y, Pritchard WF (2012). Radiopaque drug-eluting beads for transcatheter embolotherapy: experimental study of drug penetration and coverage in swine. J Vasc Interv Radiol.

[38] Burrel M, Reig M, Forner A, Barrufet M, de Lope CR, Tremosini S (2012). Survival of patients with hepatocellular carcinoma treated by transarterial chemoembolisation (TACE) using Drug Eluting Beads. Implications for clinical practice and trial design. J Hepatol.

[39] Malagari K, Pomoni M, Moschouris H, Bouma E, Koskinas J, Stefaniotou A (2012). Chemoembolization with doxorubicin-eluting beads for unresectable hepatocellular carcinoma: five-year survival analysis. Cardiovasc Intervent Radiol.

[40] Geschwind JF. Doxorubicin eluting bead sizes 100–300 μm and 70–150 μm in the VX2 model. Manuscript submitted, presented as a late breaking abstract at the Society of Interventional Radiology Meeting 2013. 2013.

[41] Hong K, Khwaja A, Liapi E, Torbenson MS, Georgiades CS, Geschwind JF (2006). New intra-arterial drug delivery system for the treatment of liver cancer: preclinical assessment in a rabbit model of liver cancer. Clin Cancer Res.

[42] Geschwind JF. Doxorubicin-eluting LC Bead M1 for patients with hepatocellular carcinoma (DEBDOX). NCT 02997954. 2015.

[43] Gonzalez MV, Tang Y, Phillips GJ, Lloyd AW, Hall B, Stratford PW (2008). Doxorubicin eluting beads-2: methods for evaluating drug elution and in vitro:in vivo correlation. J Mater Sci Mater Med.

[44] Namur J, Citron SJ, Sellers MT, Dupuis MH, Wassef M, Manfait M, Laurent A (2011). Embolization of hepatocellular carcinoma with drug-eluting beads: doxorubicin tissue concentration and distribution in patient liver explants. J Hepatol.

[45] Spreafico C, Cascella T, Facciorusso A, Sposito C, Rodolfo L, Morosi C (2015). Transarterial chemoembolization for hepatocellular carcinoma with a new generation of beads: clinical-radiological outcomes and safety profile. Cardiovasc Intervent Radiol.

[46] Kelly LR, Tatum C, Metzger T, Martin RC. Small (70–150 micron) Irinotecan drug eluting beads treatment in the management of hepatic metastatic disease: Clinical rationale and pilot data. Abstract from SIR 2011.Abstract # 242, 30 Mar 2011.

[47] Akinwande OK, Philips P, Duras P, Pluntke S, Scoggins C, Martin RC (2015). Small versus large-sized drug-eluting beads (DEBIRI) for the treatment of hepatic colorectal metastases: a propensity score matching analysis. Cardiovasc Intervent Radiol.

[48] Padia SA, Shivaram G, Bastawrous S, Bhargava P, Vo NJ, Vaidya S (2013). Safety and efficacy of drug-eluting bead chemoembolization for hepatocellular carcinoma: comparison of small-versus medium-size particles. J Vasc Interv Radiol.

[49] Heithaus RE, Cura M, Marashi KB, Sacks JD, Meler JD (2015). Abstract 142: transarterial chemoembolization with smaller beads: midterm clinical outcomes. J Vasc Interv Radiol.

[50] Rao S, Valliappan S (2015). Network S-PH. Abstract 144: feasibility of performance of drug-eluting bead transarterial chemoembolization (DEB-TACE) for hepatocellular carcinoma (HCC) as an outpatient procedure using 70-150 um microspheres. J Vasc Interv Radiol.

[51] Venkat SR, Shah MB, Barbery KJ, Checkver A, Abrahams B, Kang K (2015). Abstract 141: comparison of 70-150um (M1) versus 100-300um doxorubicin drug eluting beads in transarterial chemoembolization for hepatocellular carcinoma. J Vasc Interv Radiol.

[52] Baylatry M-T, Pelage JP, Lacombe P, Wassef M, Ghegediban HS, Lewis A, et al. Plasmatic and lung concentrations after irinotecan-eluting beads (DEBIRI) embolization of pulmonary artery alone vs. combined with bronchial artery embolization in a sheep model. CIRSE 2010. Valencia2010. p. 333.

[53] Eyol E, Boleij A, Taylor RR, Lewis AL, Berger MR (2008). Chemoembolisation of rat colorectal liver metastases with drug eluting beads loaded with irinotecan or doxorubicin. Clin Exp Metastasis.

[54] Forster REJ, Small S, Tang Y, Heaysman CL, Lloyd AW, Macfarlane WM (2010). Comparison of DC Bead-irinotecan and DC Bead-topotecan drug eluting beads for use in locoregional drug delivery to treat pancreatic cancer. J Mater Sci Mater Med.

[55] Lewis AL, Taylor RR, Hall B, Gonzalez MV, Willis SL, Stratford PW (2006). Pharmacokinetic and safety study of doxorubicin-eluting beads in a porcine model of hepatic arterial embolization. J Vasc Interv Radiol.

[56] Namur J, Citron SJ, Dupuis M, Sellers MT, Wassef M, Manfait M (2009). Diffusion of Doxorubicin from Drug Eluting Beads and Tissular Changes After Embolisation of Hepatocellular Carcinoma. J Vasc Interv Radiol.

[57] Namur J, Wassef M, Millot JM, Lewis AL, Manfait M, Laurent A (2010). Drug-eluting beads for liver embolization: concentration of doxorubicin in tissue and in beads in a pig model. J Vasc Interv Radiol.

